# Synapses mediate the effects of different types of stress on working memory: a brain-inspired spiking neural network study

**DOI:** 10.3389/fncel.2025.1534839

**Published:** 2025-03-19

**Authors:** Chengcheng Du, Yinqian Sun, Jihang Wang, Qian Zhang, Yi Zeng

**Affiliations:** ^1^Brain-inspired Cognitive Intelligence Lab, Institute of Automation, Chinese Academy of Sciences, Beijing, China; ^2^School of Future Technology, University of Chinese Academy of Sciences, Beijing, China; ^3^Center for Long-term Artificial Intelligence, Beijing, China; ^4^School of Artificial Intelligence, University of Chinese Academy of Sciences, Beijing, China; ^5^Center for Excellence in Brain Science and Intelligence Technology, Chinese Academy of Sciences, Shanghai, China

**Keywords:** short-term (working) memory, short-term plasticity, acute stress, chronic stress, rat, SNN

## Abstract

Acute stress results from sudden short-term events, and individuals need to quickly adjust their physiological and psychological to re-establish balance. Chronic stress, on the other hand, results in long-term physiological and psychological burdens due to the continued existence of stressors, making it difficult for individuals to recover and prone to pathological symptoms. Both types of stress can affect working memory and change cognitive function. In this study, we explored the impact of acute and chronic stress on synaptic modulation using a biologically inspired, data-driven rodent prefrontal neural network model. The model consists of a specific number of excitatory and inhibitory neurons that are connected through AMPA, NMDA, and GABA synapses. The study used a short-term recall to simulate working memory tasks and assess the ability of neuronal populations to maintain information over time. The results showed that acute stress can enhance working memory information retention by enhancing AMPA and NMDA synaptic currents. In contrast, chronic stress reduces dendritic spine density and weakens the regulatory effect of GABA currents on working memory tasks. In addition, this structural damage can be complemented by strong connections between excitatory neurons with the same selectivity. These findings provide a reference scheme for understanding the neural basis of working memory under different stress conditions.

## 1 Introduction

As a high-level cognitive cortex, the prefrontal cortex can perform various tasks, such as working memory, rule processing, and concept expression (Miller and Cohen, 2001). Working memory refers to the temporary storage of information in the short term, which is essential for decision-making and plays an important role in cognitive tasks (Baddeley, 1992; Cowan, 2008). When animals are engaged in working memory tasks, continuous neural activity can be observed (D'Esposito, 2007; Miller, 2013; Durstewitz et al., 2000), and fuster first showed that a single neuron in the monkey prefrontal cortex showed continuous activity throughout the delay period of a delayed response task (Fuster, 1973). Meyer et al. (2011) trained monkeys to perform behavioral tasks of spatial location and feature working memory. In addition to the ability of neurons to maintain discharge, the capacity and accuracy of working memory can also directly affect the performance of cognitive tasks (Engle, 2010). Prefrontal cortex is crucial to determining the capacity of working memory (Barbey et al., 2013). In most human subjects, working memory capacity is typically between three and six items (Cowan, 2010). Working memory capacity can be expanded through training, and human imaging studies and neurophysiological recordings in non-human primates and computational modeling studies have shown that training increases the activity of prefrontal neurons and the strength of connections between the prefrontal cortex and the parietal cortex (Constantinidis and Klingberg, 2016). However, working memory ability is not constant and is also affected by stress.

Stress hormones have significant effects on cognition and mood (Christoffel et al., 2011). Therefore, it is crucial to understand the synaptic basis behind its behavior in the brain (Shansky and Lipps, 2013; Cerqueira et al., 2007). The prefrontal cortex is particularly sensitive to stress (Robbins and Arnsten, 2009), and its internal microcircuitry can inhibit irrational reactions even under optimal conditions (Musazzi et al., 2015). Additionally, specific neurotransmitter effects influence changes in working memory capacity. Acute stress triggers NMDAR- and AMPAR-mediated increases in synaptic currents in prefrontal cortex pyramidal neurons via glucocorticoid receptors (GR), thereby enhancing firing activity and improving working memory capacity (Musazzi et al., 2010; Yuen et al., 2009). Chronic stress can lead to the loss of postsynaptic spines and apical dendrites in rodent prefrontal cortex layer 2/3 pyramidal cells (Witztum et al., 2023; Radley et al., 2004, 2006; Shansky et al., 2009; Cook and Wellman, 2004). Repeated restraint stress affects the morphology of pyramidal neurons in the rat medial prefrontal cortex (Moda-Sava et al., 2019). In the acute phase, glucocorticoid stress hormones increase the excitability of prefrontal pyramidal cells by enhancing glutamatergic synaptic transmission through increased presynaptic release and postsynaptic AMPA and NMDA receptor trafficking (Popoli et al., 2012). However, chronic exposure to excessive glucocorticoids may compensate for this increased excitability by inducing a net loss of dendritic spines (Liston and Gan, 2011), disrupting local connections and altering dynamic interactions between pyramidal cells and interneurons. This ultimately reduces the inhibitory input to pyramidal cell dendrites, impairing working memory and disrupting the normal functioning of prefrontal microcircuits (Witztum et al., 2023). Chronic behavioral stress can impair both the function and structure of the prefrontal GABAergic network, significantly disrupting GABAergic neurotransmission (Czéh et al., 2018). Compared to anatomical experimental evidence, constructing a complementary dynamic framework through biocomputational modeling to understand the interactions between different receptor types and their role in neural network behavior under stress is crucial. Some experiments have shown a significant relationship between recent life stress and reduced working memory capacity (Shields et al., 2019; Klein and Boals, 2001). However, the synaptic plasticity mechanisms that lead to this decrease in capacity are not fully understood.

Working memory performance is reflected in the ability to maintain information within a population of neurons, as well as in terms of working memory capacity, which refers to the number of items that can be processed simultaneously. Prefrontal cortex is capable of handling multiple tasks simultaneously, owing to neurons with mixed selectivity (Miller, 2013; Manohar et al., 2019). This suggests that the brain can use the same pool of neurons to selectively respond to different external inputs (Xie et al., 2022). For example, category-sensitive neurons can simultaneously distinguish between cats and dogs, and sports cars and sedans, rather than classifying them separately. Experimental evidence suggests that the maintenance of an item in working memory is achieved by the sustained activity of selective neural components in the cortex. Rolls et al. (2013) used synaptic connections in the prefrontal cortex to simulate an attractor network within a local cortical network, thereby increasing the number of short-term memory representations that can remain active simultaneously. Mi et al. (2017), based on synaptic theory, adjusted the number of items retained in working memory through external stimuli. Kim (2021) developed a spiking recurrent neural network model that successfully completed the working memory delayed matching sample (DMS) task. The temporal characteristics of the model closely resemble biological data, and enhancing the inhibitory-inhibitory connections within the network structure can improve task performance. These findings offer valuable insights for the development of working memory networks.

This study aims to investigate the mechanisms underlying the effects of acute and chronic stress on working memory performance through computational modeling, and to validate existing biological conclusions. By incorporating existing biological experimental results from rodents under acute and chronic stress, we constructed a brain-inspired spiking neural network with short-term synaptic plasticity. Specifically, we developed a rodent prefrontal neural network model with heterogeneity, driven by *in vivo* biological data, and a homogeneous network composed of random parameters as a control, to perform working memory tasks under different stress conditions. Heterogeneous networks are defined as networks where the values assigned to the network elements (such as connection probabilities, and neuronal properties) are drawn from a Gaussian distribution, informed by biological data. This approach allows for the incorporation of biological variability and complexity, better reflecting the diversity and organization observed in biological neural networks.Homogeneous networks are defined as networks where values are assigned uniformly, using random numbers. There are no distinctions made between neuron types, and all elements of the network are treated equally in terms of their parameters. The heterogeneous network integrates in vivo biological data from rodents, including the connection probabilities of neurons in layer 2/3 of the prefrontal cortex, the proportions of different types of neurons in the layer, neuronal electrophysiological parameters, and the number of dendritic spines. The results demonstrate that our heterogeneous network model improves the ability of neurons to maintain information under acute stress, which is attributed to the enhancement of AMPA and NMDA synaptic currents. This experimental result is consistent with the findings of Yuen et al. (2009). In contrast, the loss of dendritic spines in the chronic stress network impairs the ability of GABA currents to regulate working memory capacity; however, this structural loss can be compensated by intra-group excitatory connections. At the same time, both acute and chronic stress networks with heterogeneous parameters exhibit greater time scale and parameter regulation capabilities compared to homogeneous networks, suggesting that rodent biological data enhances performance in working memory tasks. In general, our study reveals how different stress states affect synaptic modulation and memory retention and clarifies the role of rodent biological information in the network.

## 2 Results

### 2.1 Working memory experimental paradigm

**Figure 1:**
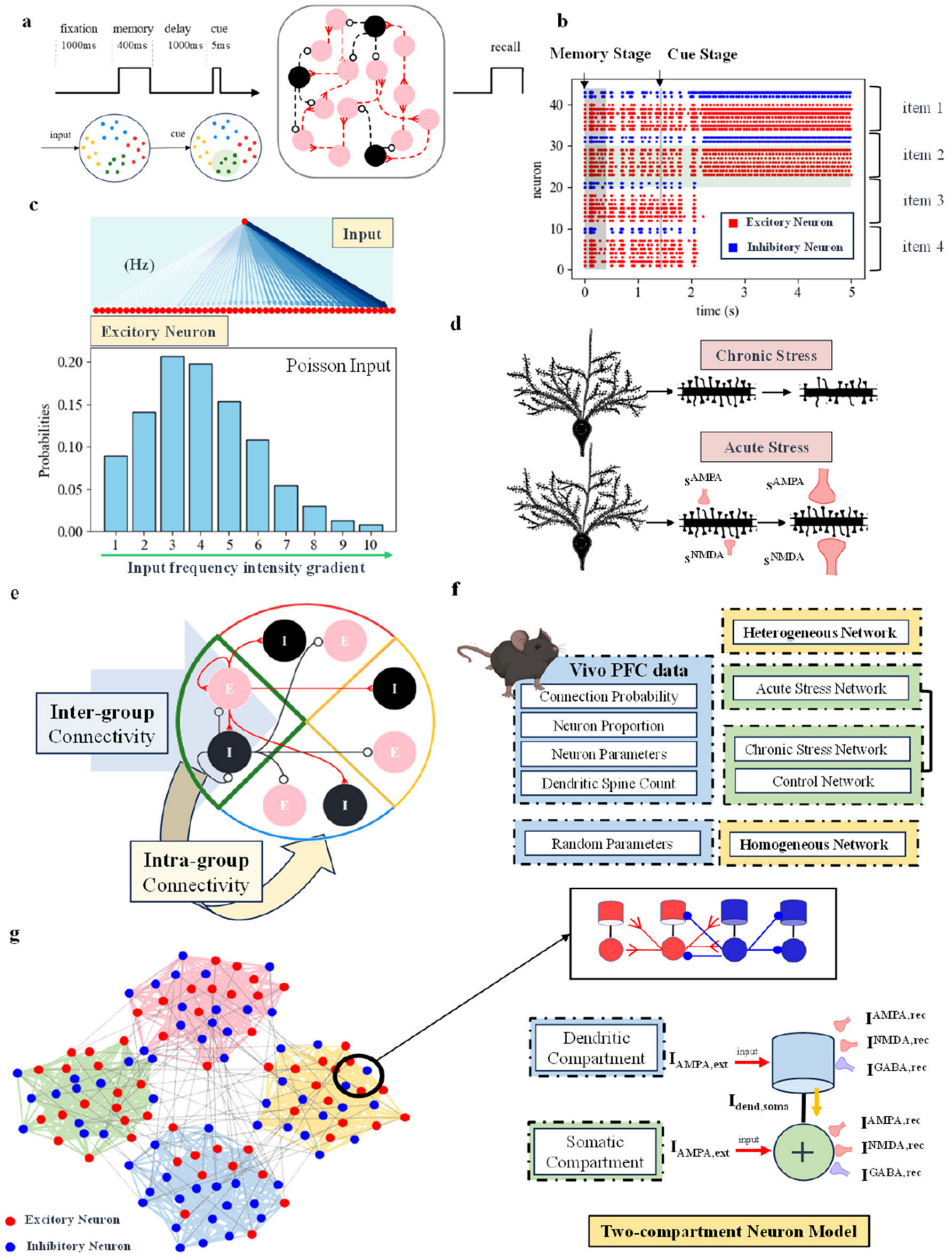
**(A)** Working memory short-term recall paradigm (Shields et al., 2019). The network includes a 400-ms memory period, a 1,000-ms delay, followed by a 5-ms short stimulus to test the network's memory. **(B)** Schematic of spike discharge in the spiking neural network. Red dots represent excitatory neurons, and blue dots represent inhibitory neurons. The figure shows 4 groups of neural populations capable of memory, with only two groups successfully maintaining discharge. It represents a scenario where the connectivity strength between excitatory neurons is enhanced under chronic stress conditions. **(C)** Schematic of network input. All excitatory neurons receive simultaneous input, with the input frequency intensity gradient following a Poisson distribution. **(D)** Schematic of computational modeling for acute and chronic stress. **(E)** Schematic of the connections between excitatory and inhibitory neuron groups within and between the groups. **(F)** The network types used in this study are divided into two categories: heterogeneous networks based on biological data from the 2/3 layer of the rodent prefrontal cortex, and homogeneous networks with random parameters. For specific sources of biological data, refer to the Methods Section. **(G)** On the left, a general schematic of the four neuronal groups in the network. The specific connections between two-compartment neurons are shown in the upper right. The lower right panel shows a schematic of the synaptic currents received by two-compartment neurons, including AMPA and NMDA excitatory synaptic currents, as well as GABA inhibitory synaptic currents.

To study the mechanism of stress on working memory, we used a spiking neural network for modeling and analysis. Given the diverse and complex nature of actual stress behavioral experiments, we approached the analysis from the perspective of neural activity, using a simple short-term working memory recall paradigm, as shown in Figure 1A. This task paradigm begins with a 1-second fixation period, followed by a 400-ms long-term input stimulus (memory period), a 1,000-ms delay (intermediate delay period), and a 5-ms cue stimulus (cue prompt period). If a neuronal group can maintain discharge when the stimulus input to the network is applied, it indicates successful memory of the stimulus information. The input to the network involves simultaneously stimulating all excitatory neurons, with inputs following a Poisson distribution, as shown in Figure 1C. The intensity of the input stimulus is determined by frequency and changes according to a specified gradient.

The theoretical maximum memory capacity of our spiking neuron cortical network model is 4, corresponding to 4 distinct groups of neurons. As shown in Figure 1B, each group consists of excitatory neurons (red) and inhibitory neurons (blue), with the central blue region serving as the boundary between groups. In the figure, in addition to excitatory neurons, inhibitory neurons also exhibit continuous discharge in the groups that are capable of sustained activity. The fact that only two groups of neurons fired continuously suggests that the inhibitory neurons suppress the activity of the other groups of neurons. The figure shows that a maximum of two neuron groups can discharge continuously, indicating that the network's current memory capacity is 2. The network connection structure sets up four different neuron groups. When one of the groups discharges continuously, it means that the memory is successful, and the network memory capacity increases by one. The network's current memory capacity is 2 because, in its steady state, only two neuron groups can discharge continuously. The remaining two groups are suppressed by GABA currents, preventing them from firing.

Based on the existing working memory experimental paradigm and network structure, we model acute and chronic stress separately. As shown in Figure 1D, from a computational modeling perspective, the characteristics of acute and chronic stress are distinguished by the structure of dendritic spines and synaptic currents. In the neuronal model, the number of dendritic spines and the three synaptic currents AMPA, NMDA, and GABA are modeled, with changes in these characteristics based on real stress experiments used to simulate the effects of stress.

### 2.2 Network structure

Animals exhibit synaptic organization principles that group neurons in specific ways (Perin et al., 2011; Pals et al., 2020). These components combine in various ways to form unique neural circuits (Bouchacourt and Buschman, 2019). Our network model represents “capacity” in the form of neuronal groups. All excitatory neurons receive input simultaneously, but due to differences in the organizational structure of synaptic connections, they exhibit varying capabilities for maintaining discharge. The specific neuronal group connection relationship is shown in Figure 1E. Each group has excitatory neurons and inhibitory neurons, which are connected to each other within the group and to neurons outside the group. Individual neuronal groups follow specific connection probabilities, are not fully interconnected, and exhibit variable connection strength. As shown in Figure 1G, the connection strength of neurons within a group is higher than that of neurons between groups. When all excitatory neurons receive external Poisson stimulation, this synaptic organization results in some neuronal groups maintaining discharge while others do not. The number of groups that can maintain discharge defines the network's current memory capacity (the maximum number of items that can be remembered). During the stimulation window, excitatory neurons within the same group facilitate each other's firing, while inhibitory neurons within the group actively suppress the firing of neurons in other groups. The network consists of four groups of selective neurons, but not all groups can maintain information simultaneously. Furthermore, they can be inhibited by inhibitory neurons from other groups. Thus, the ability to maintain information concurrently is influenced by the network structure and synaptic strength.

To model acute and chronic stress, the neuron model uses a two-compartment structure. The specific connection relationships between the two compartments are shown in Figure 1G. From a computational modeling perspective, both the dendritic and somatic compartments receive external information. In our two-compartment model, the dendrite receives excitatory and inhibitory synaptic currents from the external environment, which are transmitted through the axon and attenuated before reaching the soma. The soma integrates these currents from the dendrites and the external input to support its discharge activity.

To model the impact of acute and chronic stress on working memory tasks, we developed four distinct network models, as shown in Figure 1F. These models are generally divided into two categories: one based on a heterogeneous network using neural data from the 2/3 layer of the rodent prefrontal cortex (Hass et al., 2016), and the other a homogeneous network built with random parameters. The biological data for layer 2/3 of the mouse prefrontal cortex are comprehensive and closely related to the primary function (Shrestha et al., 2015) of working memory in mice. In addition, chronic stress has been shown to significantly affect this layer, leading to significant changes in dendritic structure. Based on the heterogeneous network structure, we created models for acute stress, chronic stress, and a control network. The heterogeneous network incorporates biological data, such as the connection probability between neurons, the distribution of pyramidal neurons and interneurons, neuronal electrophysiological parameters, and the average number of dendritic spines in the prefrontal cortex, as detailed in the Methods Section. Some parameters of the homogeneous network are derived from existing working memory models (Brunel and Wang, 2001; Rolls et al., 2013). Specifically, for acute stress, we used both the acute stress heterogeneous and homogeneous networks, while for chronic stress, we used the chronic stress heterogeneous network, the control heterogeneous network, and the homogeneous network. Since we only simulated the effect of acute stress on excitatory synaptic currents, the control network with a scaling factor set to 1 can be directly incorporated into the acute stress heterogeneous network without needing to re-establish the control network.

### 2.3 Increased AMPA and NMDA current intensity during acute stress enhances working memory capacity

**Figure 2:**
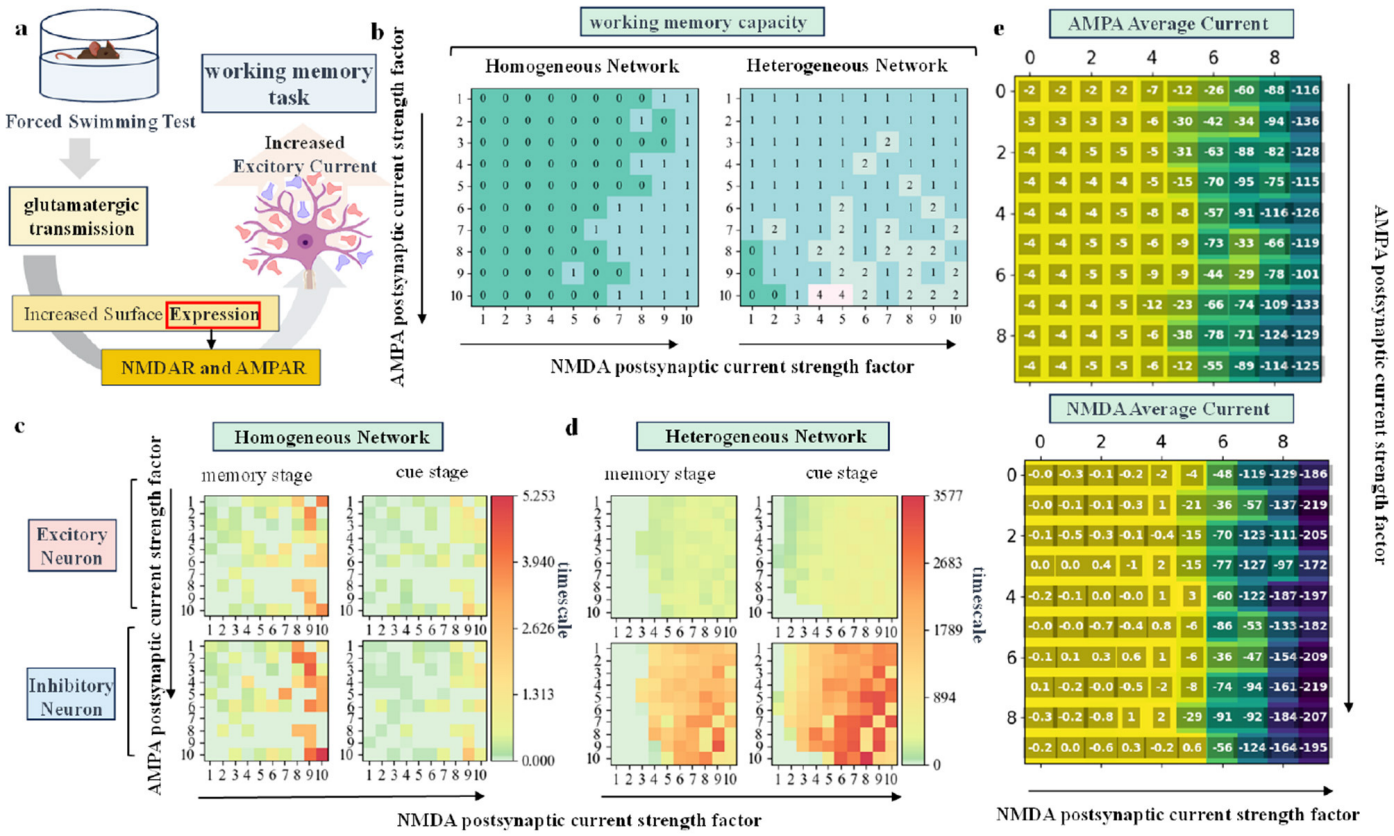
**(A)** Schematic diagram showing the effect of glutamate on working memory under swimming stress in rodent. **(B)** Distribution of working memory capacity in homogeneous and heterogeneous networks as the fractional strength of AMPA and NMDA channels changes. **(C, D)** Time scale distribution of excitatory and inhibitory neurons in homogeneous and heterogeneous networks during the memory and cue stages, respectively, as the two variable factors are altered. **(E)** Distribution of the mean AMPA and NMDA current magnitudes of excitatory neurons in the network.

Previous research has demonstrated that activation of the cortical glutamate system can enhance working memory under acute stress (Kim, 2021; Popoli et al., 2012). However, how can the primary mechanisms by which glutamatergic activity affects working memory be quantified? What changes in network dynamics induced by acute stress promote working memory? As shown in Figure 2A, *in vivo* stressors act on pyramidal neurons via glucocorticoid receptors (GR), enhancing AMPA and NMDA synaptic currents in the prefrontal cortex (Kim, 2021; Yuen et al., 2011). Various types of acute stress, such as swimming for 20 min (Roche et al., 2003), confinement in a cubicle for 2 h (Mitra et al., 2005), placement on an elevated platform for 20 min (Xu et al., 1997), or a single cortical injection of ketamine (Yuen et al., 2009), have been shown to significantly enhance NMDAR-EPSC and AMPAR-EPSC activity in prefrontal cortical pyramidal neurons, thereby improving working memory capacity. Using forced swimming stress as an example (descriptions of the remaining three conditions are detailed in the Supplementary material), acute stress significantly enhanced NMDAR-EPSC (control: 197 ≤ 15 pA, *n* = 14; swim stress: 425 ≤ 20.5 pA, *n* = 15, *P* < 0.001, ANOVA) and AMPAR-EPSC (control: 58.6 ≤ 4.4 pA, *n* = 12; swim stress: 98.8 ≤ 3.7 pA, *n* = 12, *P* < 0.001, ANOVA) magnitudes in PFC pyramidal neurons (Yuen et al., 2009). To simulate this physiological process, we adjusted the ratio of AMPA to NMDA receptor-mediated excitatory postsynaptic currents in our model based on the synaptic current ratio observed in acute stress biological experiments (see Methods for details). Correspondingly, we increased the intensity of AMPA and NMDA channels and recorded network dynamics to observe working memory changes over different time scales (Geißler et al., 2023). Additionally, we conducted experiments on a homogeneous network to investigate the factors influencing biological data from rodent prefrontal layer 2/3 during stress-related working memory tasks. As shown in Figure 2E, we recorded the AMPA and NMDA current values of excitatory neurons. While these values do not perfectly match actual biological data, we observed that increasing the intensity of the NMDA channel fraction with a longer decay time constant resulted in an overall enhancement of current values.

The simulation results are shown in Figures 2B–D. Task performance is measured using two indicators: working memory capacity and time scale. By varying the channel score strength of AMPA and NMDA, we can observe the impact of these parameters on working memory through the result matrix. Specifically, the horizontal axis of each sub-plot represents increasing NMDA channel score strength, while the vertical axis represents increasing AMPA channel score strength. Figure 2B shows that both homogeneous and heterogeneous networks exhibit a significant increase in working memory capacity as the AMPA and NMDA channel score strength increases. Overall, the heterogeneous network demonstrates a higher working memory capacity than the homogeneous network, emphasizing the role of rodent biological data in enhancing working memory performance. The ability of neurons to maintain information is quantified by the time scale: the larger the time scale, the stronger the ability to maintain information. The calculation formula is provided in the Methods Section. Figures 2C, D together illustrate the time scale distribution of excitatory and inhibitory neurons during the memory and cue prompt stages as the AMPA and NMDA channel scores are varied. An increase in AMPA and NMDA channel score strength leads to a greater time scale, improving the neurons' ability to maintain information. The heterogeneous network shows a larger time scale overall compared to the homogeneous network, with a particularly notable increase in the time scale of inhibitory neurons. The enhanced discharge capacity of inhibitory neurons contributes to improved overall network performance in working memory tasks.

### 2.4 Increased GABA currents under chronic stress suppress working memory effects

**Figure 3:**
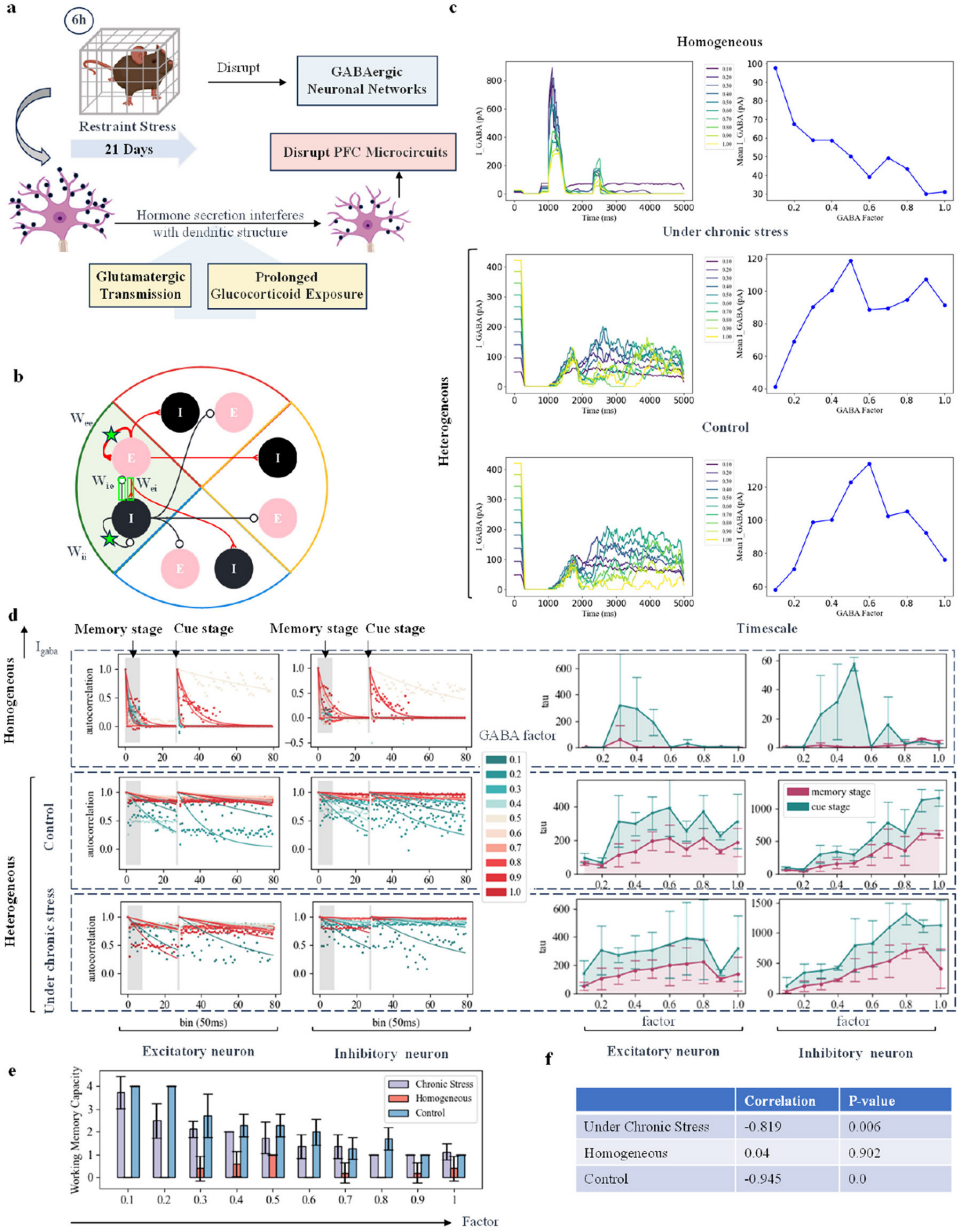
Simulating the working memory task under chronic stress by modifying inhibitory synaptic current. **(A)** Schematic diagram showing changes in neural currents and structures in rodents under chronic behavioral stress. **(B)** Schematic diagram of different excitatory-excitatory (E-E), inhibitory-excitatory (I-E), excitatory-inhibitory (E-I), and inhibitory-inhibitory (I-I) connections within the change group. Take one group as an example. **(C)** Schematic diagram of changes in neuronal currents as the fractional strength of GABA channels is adjusted. The first column shows the GABA currents of inhibitory neurons across ten groups with varying factor strengths, where colors from dark to light indicate increasing strength. The second column illustrates the changes in the average GABA current with varying intensity factors. The third column shows changes in the average AMPA and NMDA currents of excitatory neurons, with red indicating the AMPA current and green indicating the NMDA current. **(D)** Autocorrelation decay curve and time constant changes: Each sub-figure represents the dynamic changes in excitatory and inhibitory neurons. The left panel shows the autocorrelation decay curves for the memory and cue indication stages, with a color transition from green to red indicating an increase in GABA current intensity. The right panel displays the time constant changes, with red representing changes in the memory stage and green representing changes in the cue indication stage. **(E)** Network working memory capacity: This sub-figure illustrates the changes in network working memory capacity across the three models as the GABA current scaling factor is varied. **(F)** Correlation and *P* value: This sub-figure shows the correlation and *P* value between working memory capacity and changes in GABA current for each of the three models.

As shown in Figure 3A, there are various ways to induce chronic stress in animal models. Taking restraint stress as an example, rodents are subjected to daily 6-h restraint with a wire mesh for 21 consecutive days (Radley et al., 2004). This procedure leads to a significant reduction (16%) in the density of apical dendritic spines on pyramidal neurons in the medial prefrontal cortex (Radley et al., 2006).

Considering these existing experimental results, we simulated the the disruptive effects of chronic stress on GABAergic networks. In our model, we explored the impact of this disruption on working memory tasks in both homogeneous and heterogeneous networks by varying the ratio of GABA channels. We then examined strategies to compensate for this disruption. As shown in Figure 3B, we tested four approaches to modify the connection strength between neurons within a group. In this section, we begin by discussing how changes in GABAergic signaling, in the form of synaptic currents, can alter network dynamics. Biological experiments in rodents subjected to chronic stress also show changes in average GABA currents. For instance, in the control group, the average GABA BR-GIRK current was 152 ≤ 6 pA, while in the stress-adapted group, it was 141 ≤ 12 pA, and in the anhedonia group, it was 100 ≤ 5 pA. The stress-adapted group and the anhedonic group were distinguished by the sucrose consumption test. Under chronic stress, some animals exhibited resilience, with increased sucrose intake, and were classified as “stress-resilient.” In contrast, animals showing reduced sucrose intake were classified as the “anhedonic” subgroup. Figure 3C shows the schematic of average GABA current in inhibitory neurons as the GABA channel score strength changes. In the homogeneous network, increasing the GABA channel strength led to a monotonic decrease in the GABA current received by inhibitory neurons. Interestingly, in the heterogeneous network, the response followed an inverted U-shaped curve. At low GABA channel scores, increasing the channel strength enhanced the GABA current. However, as the inhibitory current reached a certain threshold, further increases in channel strength amplified inhibition of excitatory neurons, ultimately reducing the intensity of the GABA current in the inhibitory neurons.

To assess the performance of the network's working memory task under chronic stress, we used the pulse count autocorrelation decay time constant as a measure of neuronal time scale (Cavanagh et al., 2018; Wasmuht et al., 2018). A larger neuronal time scale indicates longer sustained neuronal discharge, which corresponds to better memory performance. We calculated self-decay curves under different intensity factors and plotted the time constants (time scales) of all curves separately as line graphs. The self-decay curves were calculated at two initial time points: one at the start of the memory phase and another at the start of the cue phase. If the time scale calculated from the cue phase is higher than that from the memory phase, it suggests that neurons are better able to maintain discharge during the cue phase, leading to improved memory ability. Chronic stress impaired the regulatory function of GABA currents, weakening their association with reduced working memory capacity. As shown in Figure 3D, as the GABA current increases, the firing capacity of all inhibitory neurons is significantly enhanced, and the excitatory neurons are inhibited when the intensity increases to a certain extent. In heterogeneous networks, the timescale of excitatory neurons under chronic stress conditions is generally smaller than that under normal conditions. As shown in Figure 3E, as the GABA current increased, the overall working memory capacity of the network decreased significantly. Not only was the time scale of the homogeneous network smaller than that of the heterogeneous network, but its working memory capacity was also lower. After adding rodent data to the network, both the time scale and working memory capacity increased. As shown in the table in Figure 3F, chronic stress reduced the correlation between the strength of the GABA channel score and the changes in the working memory capacity of the network.s shown in Figure 3D, the time scale of excitatory neurons in heterogeneous networks under chronic stress is smaller than under control conditions. Additionally, the time scale of homogeneous networks is smaller than that of heterogeneous networks, and their working memory capacity is also lower, as shown in Figure 3E. The inclusion of rodent data in the network increased both the time scale and working memory capacity. Chronic stress reduced the correlation between the fractional strength of the GABA channel and changes in network working memory capacity, as shown in the table in Figure 3F. Under control conditions, changes in GABA current strongly correlate with reductions in working memory capacity. However, under chronic stress, this correlation weakens, consistent with biological findings that chronic stress impairs the regulatory function of the GABAergic network.

### 2.5 Increased E-E connection strength under chronic stress is beneficial to improving working memory ability

There are various treatments for chronic stress, one of which is pharmacological intervention. Ketamine, administered at an antidepressant dose, has been shown to partially restore the baseline structure of dendritic spines in prefrontal cortex .Chronic stress induces a reduction in dendritic and synaptic density in rodent models, which is consistent with the changes in brain structure observed in patients with depression (such as MDD and PTSD) in clinical studies, especially in the hippocampus (HPC) and prefrontal cortex (PFC) regions (Koolschijn et al., 2009). This suggests that chronic stress not only changes the function of neural circuits, but also affects working memory function by changing neural structure.In network modeling, we adjusted the connection strength between neurons.Previous studies have demonstrated that stronger connections between excitatory neurons can slow down the dynamic changes of neural networks (Chaudhuri et al., 2015; Wang, 2008). Therefore, we proportionally altered the connection strength between excitatory neurons (E-E) within the same group in the network (Lam et al., 2022). We also increased the NMDA current intensity to simulate the process of ketamine treatment of chronic stress. The experimental conclusions were similar to those of increasing the intensity of excitatory neurons. The results are shown in Supplementary Figure 6. To explore these effects, we applied two input stimuli with different durations but the same intensity, representing the memory phase and the cue prompt phase, to both homogeneous and heterogeneous networks. We then analyzed the resulting changes in network time scales and working memory capacity. Furthermore, to investigate the impact of various network connections on task performance, we conducted experiments altering I-E, I-I, and E-I connections, as illustrated in Figure 3B. The detailed results of these simulations are provided in the Supplementary material.

**Figure 4:**
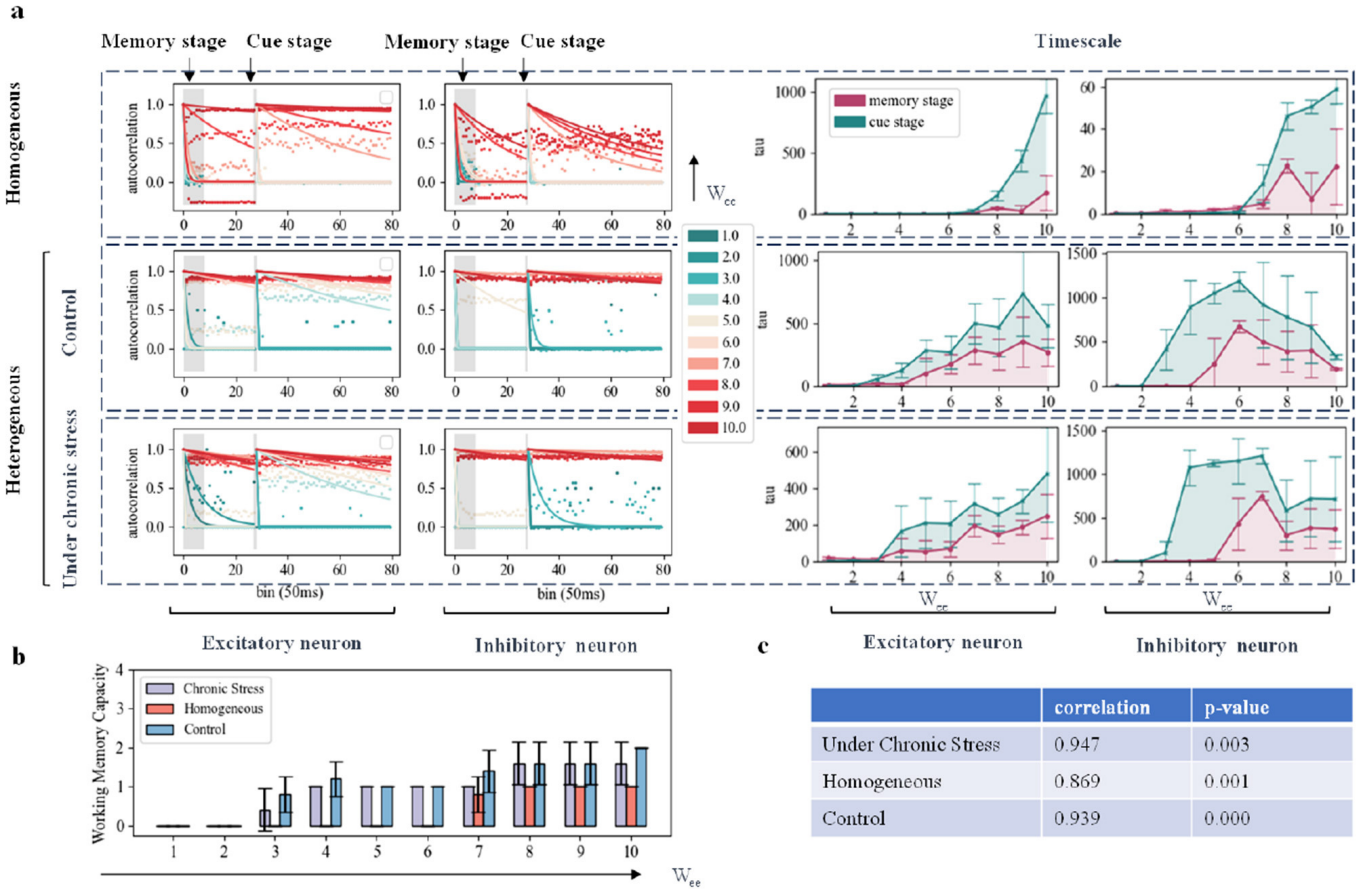
Simulation of working memory task effects under chronic stress by modifying network connection structure. **(A)** Autocorrelation Decay Curves and Time Constant Changes: Each subplot represents the dynamic changes of excitatory and inhibitory neurons. The left part displays the autocorrelation decay curves during the memory phase and cue indication phase, with colors transitioning from green to red indicating an increase in E-E connection strength. The right part shows time constant changes, with red representing changes during the memory phase and green representing changes during the cue indication phase. **(B)** Network Working Memory Capacity: This subplot illustrates the changes in network working memory capacity as a function of E-E connection strength variation by proportionality factor across the three models. **(C)** Correlation and *P* Value: The correlation and *P* value between changes in working memory capacity and E-E connection strength are shown in the three models respectively.

Figure 4A shows that in the homogeneous network model, excitatory neurons with enhanced EE connectivity exhibit a rapid increase in time scale. In heterogeneous networks, the time scale increase of excitatory neurons is more obvious. In addition, as the E-E connection strength increases, the time scale of inhibitory neurons in the heterogeneous network model changes in an inverted U shape: initially, the time scale of inhibitory neurons increases due to receiving more inputs, and the E-E connection strength increases; however, when the E-E connection strength is further enhanced, inhibitory neurons peak as a result of receiving stimulation time scales from more excitatory neurons, and inhibitory neurons with enhanced activity begin to inhibit other neurons and thus overall inhibitory neurons activity begins to decrease. This shows that rodent-specific biological information can help the network become more flexible and adaptable in the face of external input. From a time scale perspective, the reduction in synaptic density of excitatory neurons has no significant impact on network dynamics. This suggests that strong connections between excitatory neurons may compensate for the decrease in synaptic density in chronic stress models. But the results of the other three connection-changing experiments were not as significant as directly changing the connection strength between excitatory neurons, which had a more obvious positive impact on working memory performance. The specific results are shown in Supplementary Figures S3–S5. Compared with the homogeneous network, the overall working memory capacity of the heterogeneous network is higher, as shown in Figure 4B. We observed a strong correlation between increasing working memory capacity and increasing E-E connection strength in all three networks, as shown in Figure 4C. But we did not find this relationship in the I-E, II, and E-I connections. In the heterogeneous network, the impact of changes in these connections on working memory capacity was not significant. In Figures 4B, C, the working memory capacity of homogeneous networks was overall lower than that of heterogeneous networks in all cases, and the correlation between excitatory connections and working memory capacity was the lowest. This finding is consistent with Figures 3E, F. This fully demonstrates the importance of biological data for improving network working memory capacity.

## 3 Discussion

In this study, we investigated how synaptic conductance, specifically glutamatergic and GABAergic synaptic modulation, is affected in the context of stress by constructing a spiking neural network. The network consists of excitatory and inhibitory neurons interconnected through AMPA, NMDA, and GABA-conducting synapses. We simulated a short-term recall paradigm of working memory tasks under acute and chronic stress. Our network design focused on simulating the dynamics of different networks under different parameter factors, and we investigated the correlation between AMPA/NMDA currents under acute stress, GABA currents under chronic stress, and the connectivity of excitatory neurons and working memory capacity. In addition, we incorporated rodent biological information into the network to improve its overall time scale and memory capacity. This integration also enhanced the effects of specific currents and connection structures on working memory tasks.

Acute electric shock stress enhances the depolarization-induced glutamate release of presynaptic terminals in the prefrontal and frontal cortex of rats (Musazzi et al., 2010), and activates glucocorticoid receptors, increasing the transport and function of NMDAR and AMPAR via the SGK/Rab4 signaling pathway, thereby enhancing synaptic transmission and promoting PFC-mediated cognitive processes (Yuen et al., 2011). Our study investigated the effects of acute stress situations, such as forced swimming, on the function of excitatory and inhibitory neurons in simulated neural networks. We found that under these stress conditions, NMDA and AMPA currents in excitatory neurons increased, which may enhance working memory capacity. These results are consistent with previous experimental studies. In addition, when we introduced rodent information into the network, we observed that inhibitory neurons showed better long-term performance than excitatory neurons. The overall improvement in memory capacity suggests that inhibitory neurons play a crucial role in maintaining network stability and working memory function. By balancing and regulating the activity of excitatory neurons, enhanced inhibitory neuronal activity not only enhances memory capacity but also ensures overall network stability (Topolnik and Tamboli, 2022). In addition, the sustained activity of inhibitory neurons may contribute to the formation and maintenance of long-term memory , an aspect that could be explored in future work. Our results highlight the importance of incorporating animal models in neural network simulations and emphasize the important role of inhibitory neurons in cognitive function (Topolnik and Tamboli, 2022). Based on the same network structure, heterogeneous networks modeled with animal biological data have higher working memory capacity as a whole than homogeneous networks, and have stronger regulation of current and connection strength. Inhibitory neurons are able to effectively regulate the balance of network discharge. In our model, inhibitory neurons work together with excitatory neurons to balance network discharge. Specifically, in a group, inhibitory neurons inhibit the activity of neurons in other groups, thereby helping their own group to maintain continuous discharge (Deco and Rolls, 2003). This dynamic plays a key role in defining the concept of working memory capacity, where the interaction between excitatory and inhibitory neurons ensures the stability of memory maintenance.

The effects of chronic stress on working memory are multifaceted. Chronic stress-induced changes in neural circuits, such as dysfunction of the dopamine system and reward circuit, are often associated with anxiety-like and depression-like behaviors (Sanacora et al., 2022). We observed that chronic stress leads to a loss of excitatory postsynaptic spines, disrupting local connections of PFC pyramidal neurons. In chronic stress, the loss of postsynaptic spines, particularly due to dendritic atrophy of pyramidal neurons in the prefrontal cortex (PFC), damages the PFC because these dendrites are key targets for long-range excitatory cortical projections. These structural changes may compensate for reduced local glutamate release by decreasing the excitatory drive to PFC pyramidal cells. This synaptic loss on the apical dendrites can impair working memory by either affecting the activity of PFC pyramidal cells or disrupting long-range inputs from other regions, such as the hippocampus and thalamus, which are crucial for working memory processes (Bolkan et al., 2017; Witztum et al., 2023). This phenomenon may be a focus of future studies to establish the cortical and subcortical nuclei connectivity circuits involved in long-distance inputs to working memory under stress. At the same time, it reduces the frequency of spontaneous postsynaptic inhibitory currents. To simulate this process, we adjusted the proportion of GABA receptor currents in excitatory neurons in the network. Our simulation results show that increasing GABA currents contributes to an overall increase in the timescale of inhibitory neurons, but does not significantly affect the timescale of excitatory neurons. This enhanced GABA current was significantly correlated with the decrease in memory capacity, indicating that the ability of neural networks to regulate working memory through GABA currents is weakened under chronic stress.

Under simulated chronic stress conditions, it has been observed that adjusting the strength of connections between the same selective excitatory neurons (E-E) helps to offset the loss of synapse number caused by chronic stress. This enhanced E-E connection is closely associated with an increase in working memory capacity in all three network models. This suggests that while chronic stress may lead to a decrease in synaptic density of excitatory neurons, enhanced E-E connections are able to partially compensate for this loss, thereby preserving working memory function.

In future studies, it is crucial to expand the scope of research on changes in biological information under stress to include more species, such as primates and humans. Working memory differs significantly between rodents, non-human primates, and human primates, mainly in the complexity of neural mechanisms and the ability to perform working memory tasks. Another significant limitation of rodent models is that they are unable to display core symptoms of depression (such as low mood and anhedonia) due to their relatively simple brain structure (Song and Leonard, 2005). Unlike humans, nonhuman primates are able to respond to stress by producing cortisol and display core depressive-like symptoms after exposure to chronic mild stress (Qin et al., 2015). In addition, enhancing the diversity of neurons in the network, increasing the complexity of the tasks, and improving network models to closely simulate actual biological systems will allow us to gain a deeper understanding of the effects of stress on working memory. By conducting these efforts, we can more accurately simulate and predict changes in cognitive function under various stress conditions. Therefore, this will provide deeper insights and potential treatments for studying stress-related cognitive disorders. In conclusion, our study provides insights into how acute and chronic stress affects working memory, particularly at the level of neural network dynamics and synaptic connectivity. We use short-term synaptic plasticity to update the neuronal state of the working memory network, which belongs to short-term memory. In future work, we plan to further explore other synaptic plasticity mechanisms, study long-term synaptic plasticity, and study the impact on memory from multiple perspectives across brain regions and species. These findings have important implications for understanding the neurobiological basis of working memory and how stress affects cognitive function. Future studies could further explore how to use more refined neuronal morphology models to discuss the effects of stress on working memory.

## 4 Methods

### 4.1 Model architecture

Our network is a feedforward spike neuron network, in which neurons update spikes through postsynaptic currents. Specifically, we studied how the currents of other neurons affected by short-term synaptic plasticity are transmitted to the target neurons in the neuron model. This study aims to simulate different stress states by updating the network state through synaptic dynamics. The network consists of a single layer of spike neurons, and external stimuli are input to excitatory neurons through Poisson coding. Excitatory neurons stimulate the intra-group neurons and inter-group neurons connected to them. We use a short-term working memory task paradigm to record the spike discharge state of neurons updated at each time step. When the effect of changes in synaptic connections on neuronal discharge can be directly counted, the ability of neurons to maintain information changes dynamically, and the entire neuronal group as a whole shows a large amount of discharge or no discharge. The discharge of different neuronal groups constructs the concept of discrete values of working memory capacity, which is used to measure the task effect under current conditions.

### 4.2 Biological data

The connection probability and number ratio of excitatory neurons and inhibitory neurons in the network were determined using published mouse in vivo anatomical data (Hass et al., 2016), and the electrophysiological parameters of pyramidal neurons and intermediate-type neurons in the second and third layers of the rodent prefrontal cortex were selected. The effects of acute stress on mouse pyramidal neurons were achieved by exposing animals to different stressors, such as: forced swimming, acute restraint stress, elevated platform stress, and corticosterone injection (Yuen et al., 2011). Changes in neuronal AMPA and NMDA currents were caused and incorporated into the modeling as a scaling factor for synaptic currents. The effects of chronic stress on neuronal structure in mice were investigated by daily restraint stress or exposure to repeated swimming stress (Strekalova and Steinbusch, 2010), which resulted in the reorganization of the apical dendritic structure of pyramidal neurons in the rat prefrontal area (Radley et al., 2006; Cook and Wellman, 2004), reducing the density and total length of apical dendritic spines (Radley et al., 2006). In order to simulate the changes in dendrite morphology of pyramidal neurons, the neuron model uses a multi-compartment model and introduces the newly published excitatory nerves in the mouse L2 layer ACC brain area (anterior cingulate cortex) measured using 3D electron microscopy (Loomba et al., 2022). The number of spine neurons and inhibitory neurons, among which inhibitory neurons are divided into MP (multipolar) and BP (bipolar) according to their morphology (Loomba et al., 2022). According to existing literature, synapses on mouse dendritic spines are mainly excitatory (Braitenberg and Schüz, 2013), while synapses on dendritic shafts are mainly inhibitory (Karimi et al., 2020), therefore it is necessary to distinguish the number of excitatory synapses and inhibitory synapses on different types of neurons according to the proportion of inhibitory inputs to the corresponding neurons (Loomba et al., 2022). The specific value table is shown in the Supplementary material.

### 4.3 Neuron model

Each population of identically preferred neurons consists of excitatory and inhibitory populations. Our model is based on Integrate-and-Fire spiking neuron (IF) (Abbott, 1999; Hertäg et al., 2012; Zeng et al., 2023):


(1) $$\begin{array}{l} C_{m}\frac{dV\left(t\right)}{dt}=-g\left(V\left(t\right)-V_{L}\right)-I_{syn}\left(t\right) \end{array}$$

*V*denotes the subthreshold membrane potential of neurons within the ith selective population, with membrane capacitance *C*_*m*_, resting potential *V*_*L*_, and synaptic current *I*_*syn*_.

### 4.4 Synaptic current calculation

Synaptic currents include glutamatergic excitatory components mediated by AMPA and NMDA receptors, inhibitory components mediated by GABA receptors, dendritic currents, and background currents. External stimuli contribute currents through AMPA receptors. β_*NMDA*_ and β_*AMPA*_ are factors used to measure the multiples of current growth in biological experiments during acute stress conditions (actual acute stress current size/control group current size). The acute stress model in the biological model corresponds to the proportional factors of AMPA and NMDA current changes under three stress stimuli. Among them, external stimuli are input through *I*^AMPA, ext^ current. The total current is shown in the figure below:


(2) $$\begin{array}{l} I_{\text{total}}=\beta_{NMDA}I^{NMDA,rec}+I^{\text{AMPA,ext}}+\beta_{AMPA}I^{\text{AMPA,rec}}+I^{GABA}\\ \;+I^{\text{soma,dend}}+I^{\text{bg}} \end{array}$$

The number of dendritic spines affects the reception of synaptic current (Froudist-Walsh et al., 2021). We established the relationship between the number of dendritic spines and synaptic current as follows:


(3) $$\begin{array}{l} EXN=EXN\times \left(1-fac\right) \end{array}$$

(4) $$\begin{array}{l} \mu_{e}^{\text{NMDA, rec}}=EXN\times \left(1-EXN\text{\_}IN\right)\times 0.5 \end{array}$$

(5) $$\begin{array}{l} \mu_{e}^{\text{AMPA, rec}}=EXN\times \left(1-EXN\text{\_}IN\right)\times 0.5 \end{array}$$

(6) $$\begin{array}{l} \mu_{e}^{\text{GABA}}=EXN\times EXN\text{\_}IN \end{array}$$

(7) $$\begin{array}{l} \mu_{i}^{\text{NMDA, rec}}=BP\times \left(1-BP\text{\_}IN\right)\times 0.5+MP\times \left(1-MP\text{\_}IN\right)\times 0.5 \end{array}$$

(8) $$\begin{array}{l} \mu_{i}^{\text{AMPA, rec}}=BP\times \left(1-BP\text{\_}IN\right)\times 0.5+MP\times \left(1-MP\text{\_}IN\right)\times 0.5 \end{array}$$

(9) $$\begin{array}{l} \mu_{i}^{\text{GABA}}=BP\times \left(1-BP\text{\_}IN\right)\times 0.5+MP\times \left(1-MP\text{\_}IN\right)\times 0.5 \end{array}$$

Among them, *EXN* represents the average number of dendritic spines of excitatory neurons, *BP* represents the average number of dendritic spines of bipolar neurons, and *MP* represents the average number of dendritic spines of multipolar neurons. *EXN*_*I*_*N* represents the estimated value of the inhibitory input fraction [i/(i+e)] on the dendrites of mouse excitatory neurons, *BP*_*I*_*N* and *MP*_*I*_*N* represent the inhibitory input fraction values of bipolar interneurons and multipolar interneurons, respectively. When simulating the scenario of chronic stress, the percentage of dendritic spines of excitatory neurons decreased, *fac* was 0.16 in chronic stress and 0 in other cases. The specific values above are from the experimental literature (Loomba et al., 2022). The subscript *e* represents excitatory neurons, and *i* represents the value of inhibitory neurons.

The NMDA current is calculated as shown below:


(10) $$\begin{array}{l} I^{NMDA}\left(t\right)=\mu^{NMDA,rec}\frac{g^{NMDA,rec}\left(V\left(t\right)-V_{L}\right)}{1+\gamma \exp\left(-\beta V\left(t\right)\right)}\overset{N}{\underset{j=1}{\sum }}w_{j}s_{j}^{NMDA,rec}\left(t\right)u_{j}\left(t\right) \end{array}$$

μ^*NMDA, rec*^ corresponds to the proportion of the number of NMDA receptor synapses of actual excitatory pyramidal neurons in the biological model, taking the average number of excitatory synapses. *g*^*NMDA, rec*^ is the synaptic conductance of the receptor NMDA in excitatory neurons, *V*_*L*_ is the reversal potential, *s*^*NMDA, rec*^is the fraction of receptor NMDA open channels, and *w*_*j*_ is the synaptic weight. The factor *u*_*j*_ regulates excitatory synapses. γand β are constants.N is the number of neurons.

The AMPA current is calculated as shown below:


(11) $$\begin{array}{l} I^{AMPA,ext}\left(t\right)=g^{AMPA,ext}\left(V\left(t\right)-V_{L}\right)\overset{N_{ext}}{\underset{j=1}{\sum }}s_{j}^{AMPA,ext}\left(t\right) \end{array}$$

where $g_{E}^{AMPA,ext}$ is the synaptic conductance of the receptor AMPA in excitatory neurons, and *s*^*AMPA, ext*^is the fraction of receptor AMPA open channels that also receive external input. In most simulations, external EPSCs were mediated exclusively by AMPA receptors. In a few simulations, we introduced NMDA receptors in external inputs (Brunel and Wang, 2001).


(12) $$\begin{array}{l} I^{AMPA,rec}\left(t\right)=\mu^{AMPA,rec}g^{AMPA,rec}\left(V\left(t\right)-V_{L}\right)\overset{N}{\underset{j=1}{\sum }}w_{j}s_{j}^{AMPA,rec}\left(t\right)u_{j}\left(t\right) \end{array}$$

μ^*AMPA, rec*^ corresponds to the proportion of the number of AMPA receptor synapses of actual excitatory pyramidal neurons in the biological model, taking the average number of excitatory synapses. *g*^*AMPA, rec*^ is the synaptic conductance of the receptor AMPA in excitatory neurons, and *s*^*AMPA, rec*^ is the fraction of receptor AMPA open channels. N is the number of neurons.

The GABA current is calculated as shown below:


(13) $$\begin{array}{l} I^{GABA}\left(t\right)=\mu^{GABA}g^{GABA}\left(V\left(t\right)-V_{L}\right)\overset{N}{\underset{j=1}{\sum }}s_{j}^{GABA}\left(t\right) \end{array}$$

where *g*^*GABA*^ is the synaptic conductance of the receptor GABA in inhibitory neurons, and *s*^*GABA*^ is the fraction of receptor GABA open channels. N is the number of neurons.

The dendritic currents current is calculated as shown below:


(14) $$\begin{array}{l} I^{\text{dend,exc}}=I^{NMDA}+I^{\text{AMPA,ext}}++I^{\text{AMPA,rec}}+I^{\text{bg}} \end{array}$$

(15) $$\begin{array}{l} I^{\text{dend,inh}}=I^{GABA} \end{array}$$

(16) $$\begin{array}{l} I^{\text{soma,dend}}=f_{I}\left(I^{\text{dend,exc}},I^{\text{dend,inh}}\right)\\ \;=c_{1}\cdot \left[\tanh\left(\frac{I^{\text{dend,exc}}+c_{3}I^{\text{dend,inh}}+c_{4}}{c_{5}e^{-I^{\text{dend},\text{inh}}/c6}}\right)\right]+c_{2} \end{array}$$

*I*^dend,exc^ is the excitatory current flowing into the dendrites, *I*^dend,inh^is the inhibitory current flowing into the dendrites, and *I*^soma,dend^ is the current from the dendrites to the soma. c1 to c6 control the gain, translation, inversion point, and shape of the nonlinear function (Marlin and Carter, 2014).

### 4.5 Short-term synaptic plasticity

After introducing a short-term synaptic plasticity mechanism for excitatory synapses, as follows (Mi et al., 2017; Froudist-Walsh et al., 2021; Mongillo et al., 2008):


(17) $$\begin{array}{l} \frac{ds_{j}^{\text{AMPA}}\left(t\right)}{dt}=-\frac{s_{j}^{\text{AMPA}}\left(t\right)}{\tau^{AMPA}}+xu\gamma_{AMPA}\underset{k}{\sum }\delta \left(t-t_{j}^{k}\right) \end{array}$$

(18) $$\begin{array}{l} \frac{ds_{j}^{\text{NMDA}}\left(t\right)}{dt}=-\frac{s_{j}^{\text{NMDA}}\left(t\right)}{\tau^{NMDA}}+xu\left(1-s_{j}^{\text{NMDA}}\left(t\right)\right)\gamma_{NMDA}\underset{k}{\sum }\delta \left(t-t_{j}^{k}\right) \end{array}$$

(19) $$\begin{array}{l} \frac{s_{j}^{\text{GABA}}\left(t\right)}{dt}=-\frac{s_{j}^{\text{GABA}}\left(t\right)}{\tau^{GABA}}+\gamma_{I}\underset{k}{\sum }\delta \left(t-t_{j}^{k}\right) \end{array}$$

(20) $$\begin{array}{l} \frac{du}{dt}=\frac{U-u}{\tau^{u}}+U\left(1-u\right)\underset{k}{\sum }\delta \left(t-t_{j}^{k}\right) \end{array}$$

(21) $$\begin{array}{l} \frac{dx}{dt}=\frac{1-x}{\tau^{x}}-ux\underset{k}{\sum }\delta \left(t-t_{j}^{k}\right) \end{array}$$

Among them, U=0.15, τ^*u*^ = 1, 500*ms*, τ^*x*^ = 2*ms*, γ_*AMPA*_, γ_*NMDA*_ are two factors used to regulate the magnitude of the two currents under acute stress. The coefficient factor γ_*I*_ is used to regulate the GABA current and is used to change the proportion of the GABA current under chronic stress.

### 4.6 Timescale

To calculate the neuron time scale we calculated the decay time constant of the spike-count autocorrelation function for each unit during the fixation period (Murray et al., 2014). We calculated the neuron firing sequence for the first 5 seconds of the network, using the 50 ms Convert the neuronal firing sequence into a spike count matrix for a time window of unit length. Next, we calculated the Pearson correlation coefficient between the two time windows (corresponding columns of the spike count matrix) lagged Δ. Fit an exponential decay function ($\bar{\rho }$) to the Pearson correlation coefficient at each lag value using Levenberg-Marquardt (Moré, 2006; Kim, 2021) nonlinear least squares method:


(22) $$\begin{array}{l} \bar{\rho }\left(\text{Δ}\right)=a\left(\exp\left(-\frac{\text{Δ}}{\tau }\right)+b\right) \end{array}$$

where a is the fitted amplitude and b is the offset. τ is the time scale of the neuron, measuring the decay rate of the autocorrelation coefficient.

## Data Availability

The datasets presented in this study can be found in online repositories. The names of the repository/repositories and accession number(s) can be found in the article/Supplementary material.

## References

[B1] Abbott L. F. (1999). Lapicque's introduction of the integrate-and-fire model neuron (1907). Brain Res. Bull. 50, 303–304. 10.1016/S0361-9230(99)00161-6 10643408

[B2] Baddeley A. (1992). Working memory. Science 255, 556–559. 10.1126/science.1736359 1736359

[B3] Barbey A. K. Koenigs M. Grafman J. (2013). Dorsolateral prefrontal contributions to human working memory. Cortex 49, 1195–1205. 10.1016/j.cortex.2012.05.022 22789779 PMC3495093

[B4] Bolkan S. S. Stujenske J. M. Parnaudeau S. Spellman T. J. Rauffenbart C. Abbas A. I.. (2017). Thalamic projections sustain prefrontal activity during working memory maintenance. Nat. Neurosci. 20, 987–996. 10.1038/nn.4568 28481349 PMC5501395

[B5] Bouchacourt F. Buschman T. J. (2019). A flexible model of working memory. Neuron 103, 147–160. 10.1016/j.neuron.2019.04.020 31103359 PMC6613943

[B6] Braitenberg V. Schüz A. (2013). Cortex: Statistics and Geometry of Neuronal Connectivity. New York: Springer Science Business Media.

[B7] Brunel N. Wang X.-J. (2001). Effects of neuromodulation in a cortical network model of object working memory dominated by recurrent inhibition. J. Comput. Neurosci. 11, 63–85. 10.1023/A:1011204814320 11524578

[B8] Cavanagh S. E. Towers J. P. Wallis J. D. Hunt L. T. Kennerley S. W. (2018). Reconciling persistent and dynamic hypotheses of working memory coding in prefrontal cortex. Nat. Commun. 9:3498. 10.1038/s41467-018-05873-3 30158519 PMC6115433

[B9] Cerqueira J. J. Mailliet F. Almeida O. F. Jay T. M. Sousa N. (2007). The prefrontal cortex as a key target of the maladaptive response to stress. J. Neurosci. 27, 2781–2787. 10.1523/JNEUROSCI.4372-06.2007 17360899 PMC6672565

[B10] Chaudhuri R. Knoblauch K. Gariel M.-A. Kennedy H. Wang X.-J. (2015). A large-scale circuit mechanism for hierarchical dynamical processing in the primate cortex. Neuron 88, 419–431. 10.1016/j.neuron.2015.09.008 26439530 PMC4630024

[B11] Christoffel D. J. Golden S. A. Russo S. J. (2011). Structural and synaptic plasticity in stress-related disorders. Rev. Neurosci. (2011) 22:535–549. 10.1515/RNS.2011.044 21967517 PMC3212803

[B12] Constantinidis C. Klingberg T. (2016). The neuroscience of working memory capacity and training. Nat. Rev. Neurosci. 17, 438–449. 10.1038/nrn.2016.43 27225070

[B13] Cook S. C. Wellman C. L. (2004). Chronic stress alters dendritic morphology in rat medial prefrontal cortex. J. Neurobiol. 60, 236–248. 10.1002/neu.20025 15266654

[B14] Cowan N. (2008). What are the differences between long-term, short-term, and working memory? Prog. Brain Res. 169, 323–338. 10.1016/S0079-6123(07)00020-9 18394484 PMC2657600

[B15] Cowan N. (2010). The magical mystery four: how is working memory capacity limited, and why? Curr. Direct. Psychol. Sci. 19, 51–57. 10.1177/0963721409359277 20445769 PMC2864034

[B16] Czéh B. Vardya I. Varga Z. Febbraro F. Csabai D. Martis L.-S.. (2018). Long-term stress disrupts the structural and functional integrity of gabaergic neuronal networks in the medial prefrontal cortex of rats. Front. Cell. Neurosci. 12:148. 10.3389/fncel.2018.00148 29973870 PMC6020798

[B17] Deco G. Rolls E. T. (2003). Attention and working memory: a dynamical model of neuronal activity in the prefrontal cortex. Eur. J. Neurosci. 18, 2374–2390. 10.1046/j.1460-9568.2003.02956.x 14622200

[B18] D'Esposito M. (2007). From cognitive to neural models of working memory. Philos. Trans. R. Soc. B 362, 761–772. 10.1098/rstb.2007.2086 17400538 PMC2429995

[B19] Durstewitz D. Seamans J. K. Sejnowski T. J. (2000). Neurocomputational models of working memory. Nat. Neurosci. 3, 1184–1191. 10.1038/81460 11127836

[B20] Engle R. W. (2010). Role of working-memory capacity in cognitive control. Curr. Anthropol. 51, S17–S26. 10.1086/650572

[B21] Froudist-Walsh S. Bliss D. P. Ding X. Rapan L. Niu M. Knoblauch K.. (2021). A dopamine gradient controls access to distributed working memory in the large-scale monkey cortex. Neuron 109, 3500–3520. 10.1016/j.neuron.2021.08.024 34536352 PMC8571070

[B22] Fuster J. M. (1973). Unit activity in prefrontal cortex during delayed-response performance: neuronal correlates of transient memory. J. Neurophysiol. 36, 61–78. 10.1152/jn.1973.36.1.61 4196203

[B23] Geißler C. F. Friehs M. A. Frings C. Domes G. (2023). Time-dependent effects of acute stress on working memory performance: a systematic review and hypothesis. Psychoneuroendocrinology 148:105998. 10.1016/j.psyneuen.2022.105998 36493660

[B24] Hass J. Hertäg L. Durstewitz D. (2016). A detailed data-driven network model of prefrontal cortex reproduces key features of in vivo activity. PLoS Comput. Biol. 12, 1–29. 10.1371/journal.pcbi.1004930 27203563 PMC4874603

[B25] Hertäg L. Hass J. Golovko T. Durstewitz D. (2012). An approximation to the adaptive exponential integrate-and-fire neuron model allows fast and predictive fitting to physiological data. Front. Comput. Neurosci. 6:62. 10.3389/fncom.2012.00062 22973220 PMC3434419

[B26] Karimi A. Odenthal J. Drawitsch F. Boergens K. M. Helmstaedter M. (2020). Cell-type specific innervation of cortical pyramidal cells at their apical dendrites. Elife 9:e46876. 10.7554/eLife.46876 32108571 PMC7297530

[B27] Kim R. (2021). Strong inhibitory signaling underlies stable temporal dynamics and working memory in spiking neural networks. Nat. Neurosci. 24, 129–139. 10.1038/s41593-020-00753-w 33288909

[B28] Klein K. Boals A. (2001). The relationship of life event stress and working memory capacity. Appl. Cogn. Psychol. 15, 565–579. 10.1002/acp.727

[B29] Koolschijn P. C. M. van Haren N. E. Lensvelt-Mulders G. J. Hulshoff Pol H. E. Kahn R. S. (2009). Brain volume abnormalities in major depressive disorder: a meta-analysis of magnetic resonance imaging studies. Hum. Brain Mapp. 30, 3719–3735. 10.1002/hbm.20801 19441021 PMC6871089

[B30] Lam N. H. Borduqui T. Hallak J. Roque A. Anticevic A. Krystal J. H.. (2022). Effects of altered excitation-inhibition balance on decision making in a cortical circuit model. J. Neurosci. 42, 1035–1053. 10.1523/JNEUROSCI.1371-20.2021 34887320 PMC8824494

[B31] Liston C. Gan W.-B. (2011). Glucocorticoids are critical regulators of dendritic spine development and plasticity in vivo. Proc. Nat. Acad. Sci. 108, 16074–16079. 10.1073/pnas.1110444108 21911374 PMC3179117

[B32] Loomba S. Straehle J. Gangadharan V. Heike N. Khalifa A. Motta A.. (2022). Connectomic comparison of mouse and human cortex. Science 377:eabo0924. 10.1126/science.abo0924 35737810

[B33] Manohar S. G. Zokaei N. Fallon S. J. Vogels T. P. Husain M. (2019). Neural mechanisms of attending to items in working memory. Neurosci. Biobehav. Rev. 101, 1–12. 10.1016/j.neubiorev.2019.03.017 30922977 PMC6525322

[B34] Marlin J. J. Carter A. G. (2014). Gaba-a receptor inhibition of local calcium signaling in spines and dendrites. J. Neurosci. 34, 15898–15911. 10.1523/JNEUROSCI.0869-13.2014 25429132 PMC4244464

[B35] Meyer T. Qi X.-L. Stanford T. R. Constantinidis C. (2011). Stimulus selectivity in dorsal and ventral prefrontal cortex after training in working memory tasks. J. Neurosci. 31, 6266–6276. 10.1523/JNEUROSCI.6798-10.2011 21525266 PMC3103869

[B36] Mi Y. Katkov M. Tsodyks M. (2017). Synaptic correlates of working memory capacity. Neuron 93:8. 10.1016/j.neuron.2016.12.004 28041884

[B37] Miller E. K. (2013). The “working” of working memory. Dialogues Clin. Neurosci. 15, 411–418. 10.31887/DCNS.2013.15.4/emiller 24459408 PMC3898679

[B38] Miller E. K. Cohen J. D. (2001). An integrative theory of prefrontal cortex function. Annu. Rev. Neurosci. 24, 167–202. 10.1146/annurev.neuro.24.1.167 11283309

[B39] Mitra R. Jadhav S. McEwen B. S. Vyas A. Chattarji S. (2005). Stress duration modulates the spatiotemporal patterns of spine formation in the basolateral amygdala. Proc. Nat. Acad. Sci. 102, 9371–9376. 10.1073/pnas.0504011102 15967994 PMC1166638

[B40] Moda-Sava R. Murdock M. Parekh P. Fetcho R. Huang B. Huynh T.. (2019). Sustained rescue of prefrontal circuit dysfunction by antidepressant-induced spine formation. Science 364:eaat8078. 10.1126/science.aat8078 30975859 PMC6785189

[B41] Mongillo G. Barak O. Tsodyks M. (2008). Synaptic theory of working memory. Science 319, 1543–1546. 10.1126/science.1150769 18339943

[B42] Moré J. J. (2006). “The levenberg-marquardt algorithm: implementation and theory,” in Numerical Analysis: Proceedings of the Biennial Conference Held at Dundee, June 28-July 1, 1977, pages 105–116. Springer.

[B43] Murray J. D. Bernacchia A. Freedman D. J. Romo R. Wallis J. D. Cai X.. (2014). A hierarchy of intrinsic timescales across primate cortex. Nat. Neurosci. 17, 1661–1663. 10.1038/nn.3862 25383900 PMC4241138

[B44] Musazzi L. Milanese M. Farisello P. Zappettini S. Tardito D. Barbiero V. S.. (2010). Acute stress increases depolarization-evoked glutamate release in the rat prefrontal/frontal cortex: the dampening action of antidepressants. PLoS ONE 5:e8566. 10.1371/journal.pone.0008566 20052403 PMC2797327

[B45] Musazzi L. Treccani G. Popoli M. (2015). Functional and structural remodeling of glutamate synapses in prefrontal and frontal cortex induced by behavioral stress. Front. Psychiatry 6:60. 10.3389/fpsyt.2015.00060 25964763 PMC4410487

[B46] Pals M. Stewart T. C. Akyürek E. G. Borst J. P. (2020). A functional spiking-neuron model of activity-silent working memory in humans based on calcium-mediated short-term synaptic plasticity. PLoS Comput. Biol. 16:e1007936. 10.1371/journal.pcbi.1007936 32516337 PMC7282629

[B47] Perin R. Berger T. K. Markram H. (2011). A synaptic organizing principle for cortical neuronal groups. Proc. Nat. Acad. Sci. 108, 5419–5424. 10.1073/pnas.1016051108 21383177 PMC3069183

[B48] Popoli M. Yan Z. McEwen B. S. Sanacora G. (2012). The stressed synapse: the impact of stress and glucocorticoids on glutamate transmission. Nat. Rev. Neurosci. 13, 22–37. 10.1038/nrn3138 22127301 PMC3645314

[B49] Qin D. Rizak J. Chu X. Li Z. Yang S. Lü L.. (2015). A spontaneous depressive pattern in adult female rhesus macaques. Sci. Rep. 5:11267. 10.1038/srep11267 26059851 PMC4462019

[B50] Radley J. Sisti H. Hao J. Rocher A. McCall T. Hof P.. (2004). Chronic behavioral stress induces apical dendritic reorganization in pyramidal neurons of the medial prefrontal cortex. Neuroscience 125, 1–6. 10.1016/j.neuroscience.2004.01.006 15051139

[B51] Radley J. J. Rocher A. B. Miller M. Janssen W. G. Liston C. Hof P. R.. (2006). Repeated stress induces dendritic spine loss in the rat medial prefrontal cortex. Cerebral cortex 16, 313–320. 10.1093/cercor/bhi104 15901656

[B52] Robbins T. W. Arnsten A. (2009). The neuropsychopharmacology of fronto-executive function: monoaminergic modulation. Annu. Rev. Neurosci. 32, 267–287. 10.1146/annurev.neuro.051508.135535 19555290 PMC2863127

[B53] Roche M. Commons K. G. Peoples A. Valentino R. J. (2003). Circuitry underlying regulation of the serotonergic system by swim stress. J. Neurosci. 23, 970–977. 10.1523/JNEUROSCI.23-03-00970.2003 12574426 PMC6741925

[B54] Rolls E. T. Dempere-Marco L. Deco G. (2013). Holding multiple items in short term memory: a neural mechanism. PLoS ONE 8:e61078. 10.1371/journal.pone.0061078 23613789 PMC3628858

[B55] Sanacora G. Yan Z. Popoli M. (2022). The stressed synapse 2.0: pathophysiological mechanisms in stress-related neuropsychiatric disorders. Nat. Rev. Neurosci. 23, 86–103. 10.1038/s41583-021-00540-x 34893785

[B56] Shansky R. M. Hamo C. Hof P. R. McEwen B. S. Morrison J. H. (2009). Stress-induced dendritic remodeling in the prefrontal cortex is circuit specific. Cerebral Cortex 19, 2479–2484. 10.1093/cercor/bhp003 19193712 PMC2742599

[B57] Shansky R. M. Lipps J. (2013). Stress-induced cognitive dysfunction: hormone-neurotransmitter interactions in the prefrontal cortex. Front. Hum. Neurosci. 7:123. 10.3389/fnhum.2013.00123 23576971 PMC3617365

[B58] Shields G. S. Ramey M. M. Slavich G. M. Yonelinas A. P. (2019). Determining the mechanisms through which recent life stress predicts working memory impairments: precision or capacity? Stress 22, 280–285. 10.1080/10253890.2018.1556635 30767585 PMC6476640

[B59] Shrestha P. Mousa A. Heintz N. (2015). Layer 2/3 pyramidal cells in the medial prefrontal cortex moderate stress induced depressive behaviors. Elife 4:e08752. 10.7554/eLife.08752 26371510 PMC4566133

[B60] Song C. Leonard B. E. (2005). The olfactory bulbectomised rat as a model of depression. Neurosci. Biobehav. Rev. 29, 627–647. 10.1016/j.neubiorev.2005.03.010 15925697

[B61] Strekalova T. Steinbusch H. W. (2010). Measuring behavior in mice with chronic stress depression paradigm. Progr. Neuro-Psychopharmacol. Biol. Psychiat. 34, 348–361. 10.1016/j.pnpbp.2009.12.014 20026369

[B62] Topolnik L. Tamboli S. (2022). The role of inhibitory circuits in hippocampal memory processing. Nat. Rev. Neurosci. 23, 476–492. 10.1038/s41583-022-00599-0 35637416

[B63] Wang X.-J. (2008). Decision making in recurrent neuronal circuits. Neuron 60, 215–234. 10.1016/j.neuron.2008.09.034 18957215 PMC2710297

[B64] Wasmuht D. F. Spaak E. Buschman T. J. Miller E. K. Stokes M. G. (2018). Intrinsic neuronal dynamics predict distinct functional roles during working memory. Nat. Commun. 9:3499. 10.1038/s41467-018-05961-4 30158572 PMC6115413

[B65] Witztum J. Singh A. Zhang R. Johnson M. Liston C. (2023). An automated platform for assessing working memory and prefrontal circuit function. Neurobiol. Stress 24:100518. 10.1016/j.ynstr.2023.100518 36970451 PMC10033752

[B66] Xie Y. Hu P. Li J. Chen J. Song W. Wang X.-J.. (2022). Geometry of sequence working memory in macaque prefrontal cortex. Science 375, 632–639. 10.1126/science.abm0204 35143322

[B67] Xu L. Anwyl R. Rowan M. J. (1997). Behavioural stress facilitates the induction of long-term depression in the hippocampus. Nature 387, 497–500. 10.1038/387497a0 9168111

[B68] Yuen E. Y. Liu W. Karatsoreos I. N. Feng J. McEwen B. S. Yan Z. (2009). Acute stress enhances glutamatergic transmission in prefrontal cortex and facilitates working memory. Proc. Nat. Acad. Sci. 106, 14075–14079. 10.1073/pnas.0906791106 19666502 PMC2729022

[B69] Yuen E. Y. Liu W. Karatsoreos I. N. Ren Y. Feng J. McEwen B. S.. (2011). Mechanisms for acute stress-induced enhancement of glutamatergic transmission and working memory. Mol. Psychiatry 16, 156–170. 10.1038/mp.2010.50 20458323 PMC3108461

[B70] Zeng Y. Zhao D. Zhao F. Shen G. Dong Y. Lu E.. (2023). Braincog: a spiking neural network based, brain-inspired cognitive intelligence engine for brain-inspired ai and brain simulation. Patterns 4:100789. 10.1016/j.patter.2023.100789 37602224 PMC10435966

