# Supplementary material for: Synapses mediate the effects of different types of stress on working memory: a brain-inspired spiking neural network study
Source: Front Cell Neurosci. 2025 Mar 19;19:1534839. doi: 10.3389/fncel.2025.1534839 (PMC11961926; doi:10.3389/fncel.2025.1534839)
Supplement: Supplementary file 1 [file Data_Sheet_1.pdf]

Supplementary Material

Chengcheng Du[a,d,e], Yinqian Sun[a,d,e], Jihang Wang[a,d,e], Qian Zhang[a,b,e,*], Yi Zeng[a,b,c,e,*]

[a] *Brain-inspired Cognitive Intelligence Lab, Institute of Automation, Chinese Academy of Sciences, Beijing, 100190, China.*
[b] *School of Artificial Intelligence, University of Chinese Academy of Sciences,Beijing, 100049, China.*
[c] *Key Laboratory of Brain Cognition and Brain-inspired Intelligence Technology, Chinese Academy of Sciences, China.*
[d] *School of Future Technology, University of Chinese Academy of Sciences, Beijing, 101408, China.*
[e] *Center for Long-term Artificial Intelligence, Beijing, China.*

[*] Correspondence: q.zhang@ia.ac.cn, yi.zeng@braincog.ai

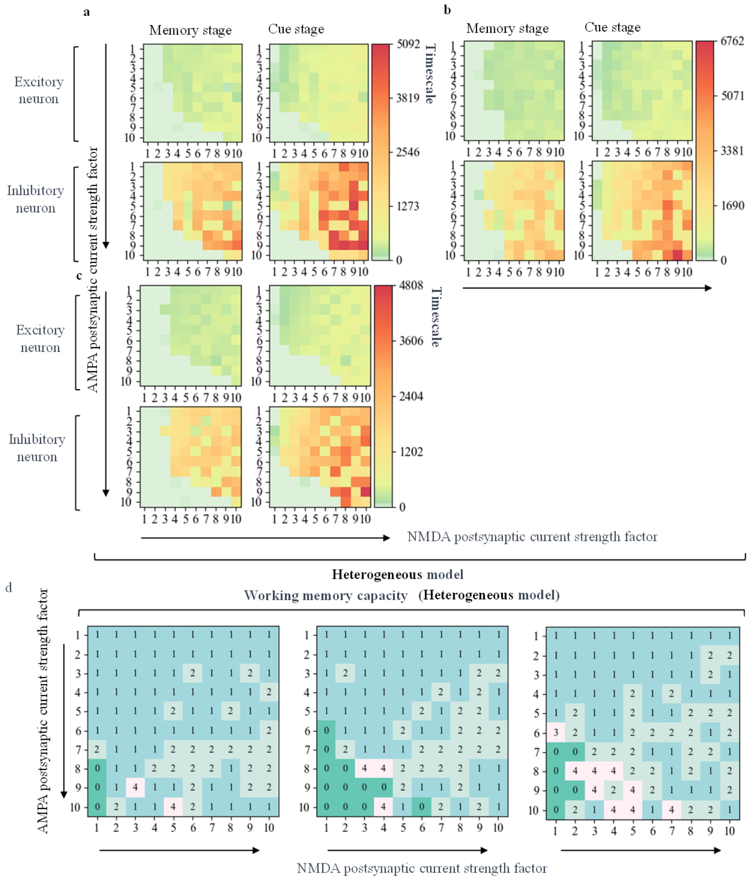

Supplementary Figure 1: **Changes in Synaptic Current Time Scale Distributions under Three Acute Stressors.** (a)(b)(c) represent the time constant distribution plots under three different conditions: (a) restraining rats in a small compartment for 2 hours[1]; (b) placing rats on an elevated platform for 20 minutes[2]; or (c) a single injection of corticosterone (which mimics acute stress-induced levels)[**?**].(d)distribution of Synaptic Currents and Working Memory Capacity under Three Acute Stressors.From left to right, they are consistent with the corresponding situations in a-c.

a

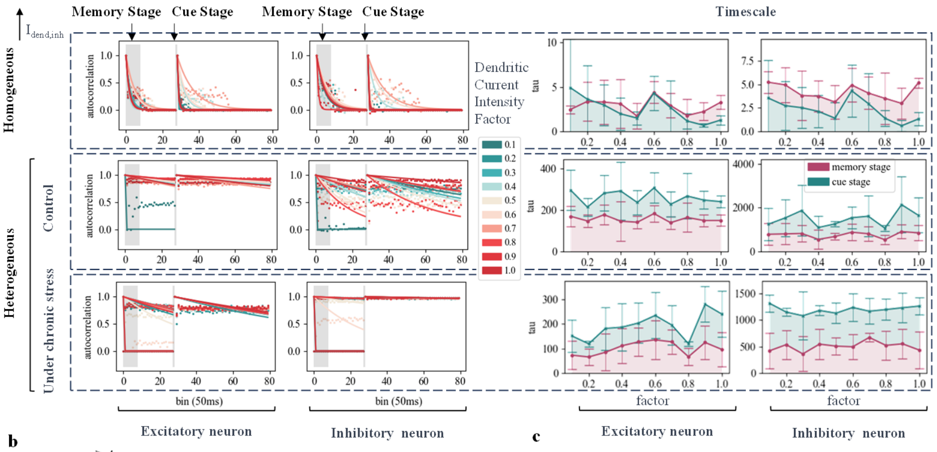

b

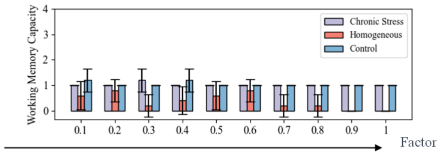

c

	correlation	p-value
Under Chronic Stress	-0.290	nan
Homogeneous	-0.686	0.028
Control	-0.522	0.121

Supplementary Figure 2: **Simulation of Working Memory Task Effects under Chronic Stress by modifying Dendritic Inhibitory Synaptic Current.**(a) Autocorrelation Decay Curves and Time Constant Changes:Each subplot represents the dynamic changes of excitatory neurons and inhibitory neurons . The left part displays the autocorrelation decay curves during the memory phase and cue indication phase, with colors transitioning from green to red indicating an increase in dend current strength. The right part shows time constant changes, with red representing changes during the memory phase and green representing changes during the cue indication phase.(b) Network Working Memory Capacity:This subplot illustrates the changes in network working memory capacity as a function of dend current variation by proportionality factor across the three models.(c) Correlation and P Value:The correlation and P value between changes in working memory capacity and dend current are shown in the three models respectively.

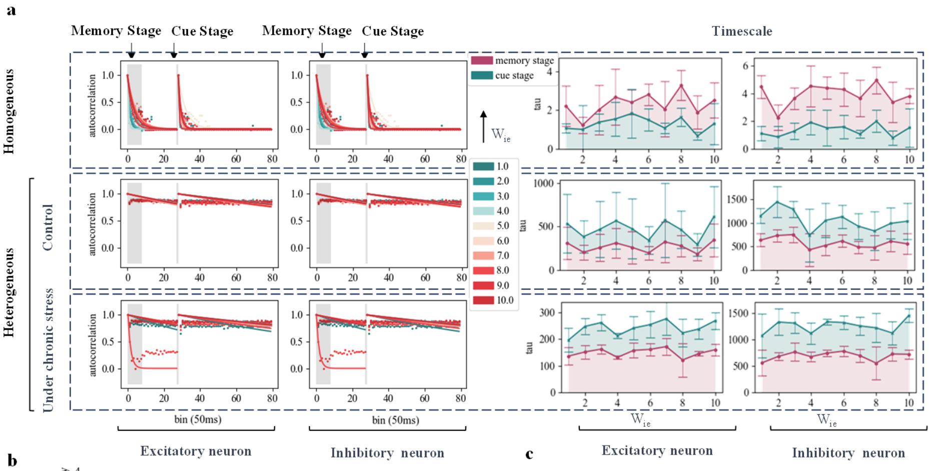

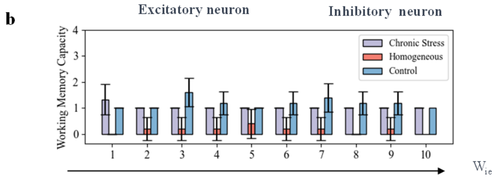

c

	correlation	p-value
Under Chronic Stress	-0.522	nan
Homogeneous	-0.174	0.631
Control	0.018	0.960

Supplementary Figure 3: **Simulation of Working Memory Task Effects under Chronic Stress by Modifying Network Connection Structure.**(a) Autocorrelation Decay Curves and Time Constant Changes: Each subplot represents the dynamic changes of excitatory and inhibitory neurons. The left part displays the autocorrelation decay curves during the memory phase and cue indication phase, with colors transitioning from green to red indicating an increase in I-E connection strength. The right part shows time constant changes, with red representing changes during the memory phase and green representing changes during the cue indication phase. (b) Network Working Memory Capacity: This subplot illustrates the changes in network working memory capacity as a function of I-E connection strength variation by proportionality factor across the three models. (c) Correlation and P Value: The correlation and P value between changes in working memory capacity and I-E connection strength are shown in the three models respectively.

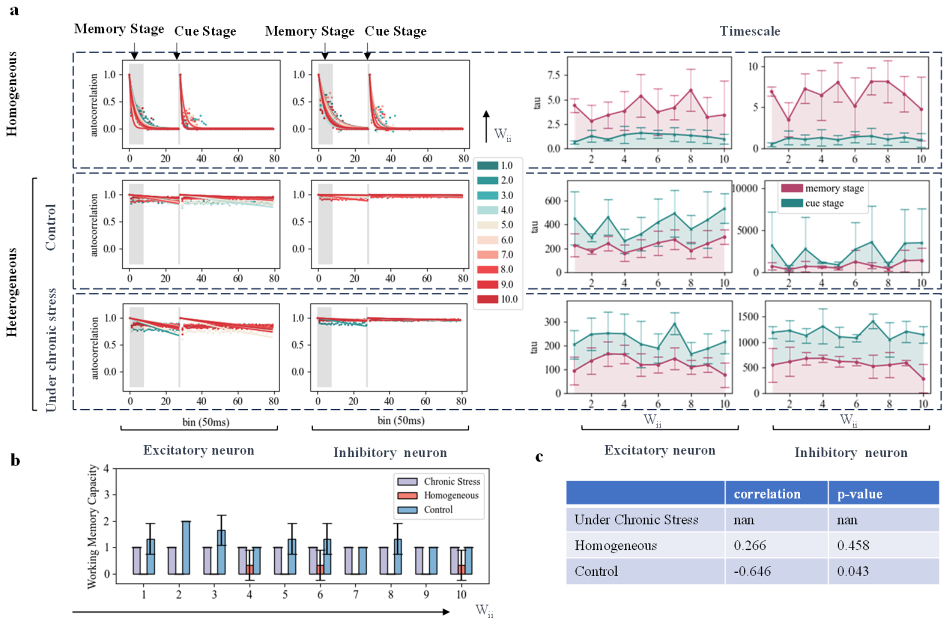

	correlation	p-value
Under Chronic Stress	nan	nan
Homogeneous	0.266	0.458
Control	-0.646	0.043

Supplementary Figure 4: **Simulation of Working Memory Task Effects under Chronic Stress by Modifying Network Connection Structure.**(a) Autocorrelation Decay Curves and Time Constant Changes: Each subplot represents the dynamic changes of excitatory and inhibitory neurons. The left part displays the autocorrelation decay curves during the memory phase and cue indication phase, with colors transitioning from green to red indicating an increase in I-I connection strength. The right part shows time constant changes, with red representing changes during the memory phase and green representing changes during the cue indication phase. (b) Network Working Memory Capacity: This subplot illustrates the changes in network working memory capacity as a function of I-I connection strength variation by proportionality factor across the three models. (c) Correlation and P Value: The correlation and P value between changes in working memory capacity and I-I connection strength are shown in the three models respectively.

a

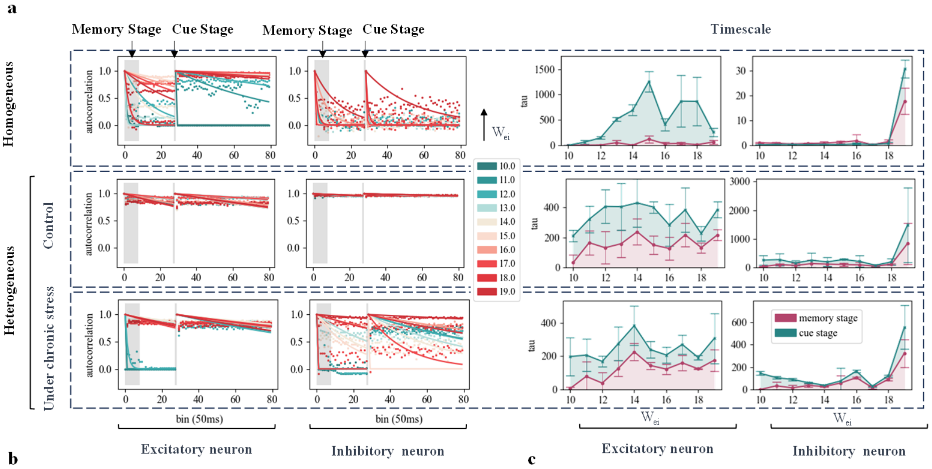

b

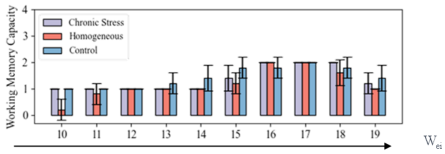

c

	correlation	p-value
Under Chronic Stress	0.686	nan
Homogeneous	0.696	0.025
Control	0.779	0.008

Supplementary Figure 5: **Simulation of Working Memory Task Effects under Chronic Stress by Modifying Network Connection Structure.**(a) Autocorrelation Decay Curves and Time Constant Changes: Each subplot represents the dynamic changes of excitatory and inhibitory neurons. The left part displays the autocorrelation decay curves during the memory phase and cue indication phase, with colors transitioning from green to red indicating an increase in E-I connection strength. The right part shows time constant changes, with red representing changes during the memory phase and green representing changes during the cue indication phase. (b) Network Working Memory Capacity: This subplot illustrates the changes in network working memory capacity as a function of E-I connection strength variation by proportionality factor across the three models. (c) Correlation and P Value: The correlation and P value between changes in working memory capacity and E-I connection strength are shown in the three models respectively.

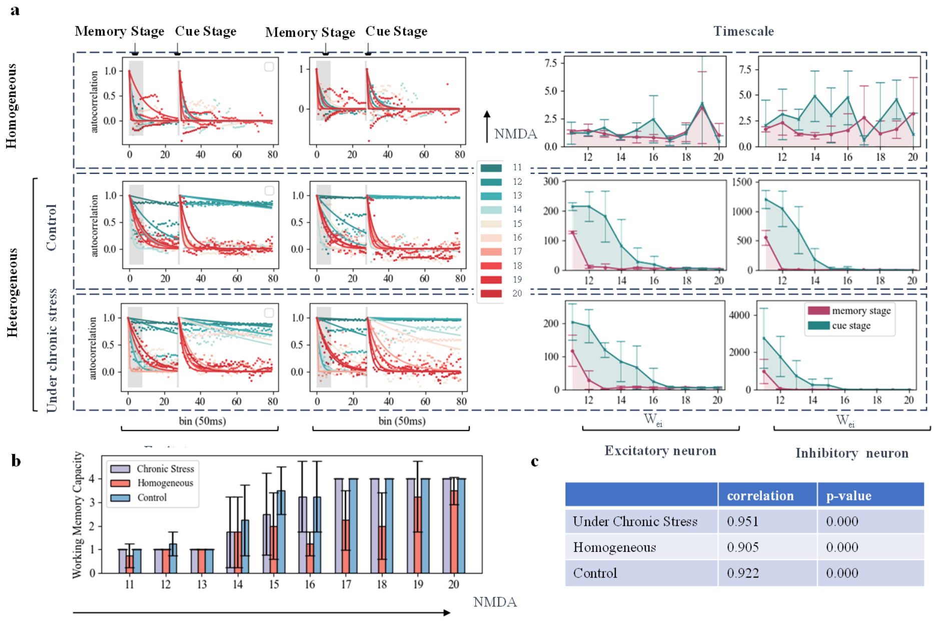

	correlation	p-value
Under Chronic Stress	0.951	0.000
Homogeneous	0.905	0.000
Control	0.922	0.000

Supplementary Figure 6: **The effect of working memory task under chronic stress was simulated by modifying the NMDA current intensity.** (a) Autocorrelation decay curve and time constant change: Each sub-figure represents the dynamic changes of excitatory and inhibitory neurons. The left part shows the autocorrelation decay curves of the memory stage and the cue indication stage, and the color changes from green to red indicate the increase in the strength of E-I connection. The right part shows the change of time constant, red represents the change in the memory stage, and green represents the change in the cue indication stage. (b) Network working memory capacity: This sub-figure illustrates the scaling factor of the network working memory capacity with the change of NMDA current intensity in the three models. (c) Correlation and P value: The three models show the correlation and P value between working memory capacity and changes in NMDA current intensity.

pre	post	p_{con}
PC L2/3	PC L2/3	0.139
PC L2/3	IN-L L2/3	0.325
PC L2/3	IN-CL L2/3	0.159
PC L2/3	IN-F L2/3	0.290
IN-L L2/3	PC L2/3	0.466
IN-CL L2/3	PC L2/3	0.301
IN-F L2/3	PC L2/3	0.710
IN L2/3	IN L2/3	0.250

Supplementary Table 1: **Connectivity between different cortical columns and between pyramidal neurons and intermediate neurons in the L2/3 layer of the rodent prefrontal cortex, denoted as** p_{con}. Where PC represents pyramidal neurons, and IN represents intermediate-type neurons. We defined local interneurons (IN-L) with projections within the same layer and column as fast-spiking cells, cross-layer interneurons (IN-CL) as bitufted cells, and far-reaching interneurons (IN-F) with projections both outside of their column and layer of origin as Martinotti cells [3].

Layer	PC	IN-L	IN-CL	IN-CC	IN-F
L 2/3	47/57.4%	3.1/57.4%	2.6/57.4%	2.6/57.4%	2.1/57.4%

Supplementary Table 2: **The ratio of the number of pyramidal neurons in the L2/3 layer of the rodent prefrontal cortex to different types of intermediate neurons.**[3].

parameter	PCL2/3	FS	BT	MC
C(pF)	164.96(59.11)	59.58(10.59)	79.36(14.83)	81.12(28.96)
g_L(nS)	7.04(1.72)	5.34(0.91)	3.99(0.51)	2.98(0.55)

Supplementary Table 3: **Neuron Parameters.** Mean and variance of membrane capacitance and leak conductance parameters for four types of neurons.(FS: Fast-spiking interneuron, BT: Bitufted interneuron, MC: Martinotti cell) [3].

	EXN	MP	BP
number (mean)	251	544	705

Supplementary Table 4: **Neuronal Dendritic Spine Counts.** Number of dendritic spines for three types of neurons: EXN: Pyramidal neurons (excitatory neurons), MP: Multipolar interneurons, BP: Bipolar interneurons. [4].

References

[1] Rupshi Mitra, Shantanu Jadhav, Bruce S McEwen, Ajai Vyas, and Sumantra Chattarji. Stress duration modulates the spatiotemporal patterns of spine formation in the basolateral amygdala. *Proceedings of the National Academy of Sciences*, 102(26):9371–9376, 2005.

[2] Lin Xu, Roger Anwyl, and Michael J Rowan. Behavioural stress facilitates the induction of long-term depression in the hippocampus. *Nature*, 387(6632):497–500, 1997.

[3] Joachim Hass, Loreen Hertäg, and Daniel Durstewitz. A detailed data-driven network model of prefrontal cortex reproduces key features of in vivo activity. *PLOS Computational Biology*, 12(5):1–29, 05 2016.

[4] S Loomba, J Straehle, V Gangadharan, N Heike, A Khalifa, A Motta, N Ju, M Sievers, J Gempt, HS Meyer, et al. Connectomic comparison of mouse and human cortex. science 377, eabo0924, 2022.
